# The Economic Rationale for Healthcare Reform

**DOI:** 10.34172/ijhpm.8441

**Published:** 2024-06-16

**Authors:** Naoki Ikegami

**Affiliations:** Keio University, Tokyo, Japan.

**Keywords:** Clinical Pathways, EQ-5D, Pharmaceuticals, Japan

## Abstract

Healthcare reform is analyzed from an economic perspective. First, the economic rationale for providing access to healthcare lies in the benefit from knowing that those without means would be able to access health services. However, this does not explain why they should be entitled to the same quality of service. In practice, even in high-income countries, patients who are willing and able to pay tend to have better access to specialist services. Secondly, the division of labor has not increased efficiency in healthcare because health services are provided by professionals who have autonomy. However, efficiency can be increased by standardizing the process with clinical pathways and shifting service delivery from physicians to nurses and technicians. Thirdly, cost-effectiveness analysis is being used to making decisions on listing pharmaceutical products in the national formulary, but pricing and prescribing have continued to be made idiosyncratically. Lastly, Japan’s healthcare system is analyzed based on this framework.

 Health for All has been the goal in health policy.^1^ To achieve this goal, stakeholders must be the engaged and empowered.^2^ However, this has been difficult to achieve. In this editorial, I examine the following policy objectives in healthcare reform from an economic perspective: achieving equal access to health service for all patients, delivering services more efficiently by the division of labor, and using cost-effective analysis for making decisions on the listing and pricing of pharmaceuticals. Finally, I describe the strengths and weaknesses of Japan’s healthcare system from which this analytic framework was conceived.

## Equal Access to Health Services

 The economic rationale for patients to have equal access to health services lies in the “informational externalities.”^3^ This is the benefit from knowing that patients without means would be able to access to health services. The goal has been expanded to not only having access, but having equal access. This means that all patients should be provided with the same quality of service. This would also be in line with the physicians’ professional commitment to provide the best service to all patients, regardless of how much they are paid.

 However, health services are not necessary delivered in line with this egalitarian principle even in high-income countries. Those who are able and willing to pay generally tend to have better access to healthcare. In England, private patients are not placed in long waiting list and may choose specialists.^4^ In France, although health services are fully covered by public health insurance, specialists are allowed to balance bill (charge extra).^5^ Moreover, patients who pay privately are treated by renowned physicians. The value of their services is not limited to their professional competence, but also from the fact that being treated by a renowned specialist has an intrinsic value, irrespective of the outcome. The value comes from the fact that their services are limited. The economic term for the value that comes from their scarcity, such as works of art, is positional goods.^6^

 Moreover, it should also be noted that there is no consensus on the amount that physicians should earn compared with other workers. There are considerable variations even among high-income countries. In Norway, physicians earn only 1.9 times the earnings of average workers but in the Netherlands, they earn 8.7 times.^7^ Such differences are the result of historical legacies and do not reflect the extent to which physicians have contributed towards improving patient outcomes.

## Increasing Efficiency

 Adam Smith explained how pins could be efficiently made by dividing the task so that “one man draws out the wire, another straights, a third cuts” and so forth.^8^ However, in healthcare, the division of labor among physicians, nurses and other health workers has increased, rather than decreased, the number of workers performing the task. Why should this be the case? In producing pins, unskilled workers were hired at low wages and trained to perform the task. The wire used as material and the pins produced were the same.

 In contrast, in healthcare, services must be tailored to meet the unique needs of the patient. Moreover, the needs are determined by physicians who have autonomy and do not take costs into consideration. There is also no agreement on whether services should be delivered only by physicians or whether the services could (should) be shifted (transferred) and/or shared with other health workers. As a result, the division of labor (services) among physicians and other health workers has increased, rather than decreased, costs. Moreover, the division of labor has also developed in the allied health professions. For example, to deliver rehabilitation services, physiotherapists, occupational therapists, and speech therapists are needed irrespective of the workload.

 Under these conditions, how can efficiency be increased in healthcare, that is the same or better outcomes at lower costs? The first is clinical pathways.^9^ Clinical pathways define the services to be delivered for each day from admission to discharge based on the patient’s diagnosis or surgical procedure. By doing so, the delivery of service is standardized and could be delivered more efficiently.

 The second is to shift the task, such as taking an electrocardiogram, from physicians to technicians. Most health services are routine and do not necessary require much judgement. The dividing line between what could or should be performed by nurses or technicians and what must be performed by physicians is based more on a historical legacy than on the technical skills required. In general, as the technology becomes more widely available, the task can be more readily shifted. Any opposition from physicians based on quality concerns could be resolved by evaluating the patient’s outcome.

 The third is to contain costs by limiting the number of specialists and medical centers. This will be the most effective way of containing costs. However, such containment would be opposed by young physicians who want to become specialists. It would also be opposed by politicians because they would like to open medical centers to show their commitment to the health of their constituents.

 However, cost-effective analysis does not provide conclusive evidence. In order to compare patient outcomes with costs, adjustments have to be made for their age and clinical conditions. Evaluating the process would be easier. However, in order to do so, medical records must be kept and reviewed by the physicians of the same specialty, which requires considerable investment of resources. This is why quality tends to be focused on the structure, such as the number of physicians and hospital beds. However, the extent to which they impact on the health of the population is difficult to evaluate.

## Evaluating Costs and Outcomes of Pharmaceuticals

 Pharmaceuticals are one of few areas where health economic evaluation is made. This is because outcomes and costs are easier to evaluate, and because pharmaceutical companies have the resources to conduct the analysis. Evaluation has been facilitated by the development of the EQ-5D.^10^ The EQ-5D is composed of five questions on mobility, selfcare, usual activities, pain/discomfort, and anxiety/depression. In each, the subject selects from five levels, that range from no problem to being unable to perform the activity or to being in the worst condition. The responses to the five questions are converted to a global score of the subject’s health status by looking up the combination in the conversion table.

 This table has been derived from a survey of the general population on how they would value the time spent in hypothetical conditions compared with the time spent in perfect health. The scale is of five levels with 1 being the best and 5 being the worst condition. For example, the EQ-5D value for a patient responding as being in level 1 in mobility, 2 in self-care, 2 in usual activities, 4 in pain, and 4 in anxiety/depression, would be 0.503 according to the survey conducted in Japan.^11^ This means that the number of days spent in this condition would be valued at about half the time spent in perfect health.

 By evaluating the quality of life with the EQ-5D and the time patients spent at each level, it is possible to compare the cost-effectiveness of a new pharmaceutical product with that of an existing product by using quality adjusted life year (QALY). QALY is calculated by summing the time spent in each health status measured by the EQ-5D. If the cost per QALY based on the proposed price of the product is below the threshold set by the government, then it would provide a strong argument to list the product in the government formulary. However, setting the price of the product has continued to be a complicated process in which other factors, such as the price of existing products for the same clinical conditions, the impact of listing on the government budget and the need to promote domestic production are considered. Parenthetically, the EQ-5D is generally not used by clinicians because it does not provide the level of details that is needed at the clinical level. For this purpose, disease-specific quality-of-life scales have been developed and has been used to promote sales after the product has been approved.

## How Costs Have Been Contained in Japan

 The above is based on my analysis of Japan’s healthcare system.^12^ Physicians are not divided into specialists and generalists because of the following decisions made by the government. The government decided to recognize only western medicine in 1871, but medical licenses were given to the existing practitioners and also their sons in 1882. This made it possible for patients to have continued access to primary care services. University level education for physicians was introduced in 1887, but the majority of physicians were trained in vocational schools until 1952. Those trained in the latter practiced mainly in clinics and rural hospitals because they found it difficult to obtain positions in the prestigious urban medical centers as they were reserved for graduates of elite universities.

 However, the income of the primary care physicians has tended to be higher than that of the specialists in medical centers. This is because when social health insurance was implemented in 1926, the Japan Medical Association set the fees. In doing so, they favored the services provided by their constituents: the physicians in clinics who delivered primary care services. Although the fee schedule has been revised, it has retained this basic structure. As a result, the fees for specialist services have continued to be set comparatively low. This is why most medical centers operate at a deficit and why high-tech services are mostly delivered by the subsidized public-sector hospitals. The physicians employed in these hospitals generally earn less than private practitioners in clinics, but they are compensated for their lower income by being able to focus on high-tech services. Thus, among physicians, professional prestige and income do not come together but tend to compensate each other.

 The second feature of Japan’s healthcare system is that pharmaceuticals are included in the fee schedule. This is because dispensing was traditionally performed by physicians and because pharmaceuticals compose one fifth of the total health expenditures and the national government finances one quarter of the total. Unlike physician services, pharmaceutical companies have been willing to lower prices so as to promote sales. Thus, healthcare providers generally make a profit from dispensing. However, the government conducts a survey of market prices and volume sold. Based on the results which tend to show market prices to be lower than the price set by the government, the government lowers the price at which the product will be reimbursed so that the revised price will be only 2 percent of its volume-weighted market price. This has led to a continuous downward spiral of prices. The price of a new product is generally set by comparing its effectiveness with a comparator and/or evaluating its innovativeness. Prices are reduced if sales exceed the amount which was estimated by the pharmaceutical company. Evaluations by the EQ-5D have been generally restricted to providing data on whether the product should be listed and not for setting its price.

 The third feature is that costs have been contained on the demand side by levying coinsurance. Initially, there was no coinsurance for employees enrolled in social health insurance because the objective lay in returning them back to the workforce as quickly as possible. However, when coverage was extended to others, a 50% coinsurance was introduced. In 1973, this coinsurance was waived for elders seventy and over and made services free. The subsequent escalation in costs have led to the present rate which is basically 30% for all except for elders who have low-income and most children. This coinsurance has made patients and providers aware of healthcare costs. However, financial protection has been provided by the introduction of catastrophic coverage in 1973 that has set a ceiling on the coinsurance. It is in this context that the government introduced a 7000 Yen ($US 50) copayment on top of the 30% coinsurance should the patient visit medical centers without referral except for emergencies. The extent to which this copayment has contained the number of patients visiting medical centers without referral has not been evaluated, nor whether adverse events have occurred among patients with low-income. However, it has sent a clear message to the public that they must obtain a referral before they visit a medical center.

## Conclusion

 Health services have become increasingly difficult to finance because of the advances in technology and the rising expectations of the public. The gap between the services that physicians decide as being needed and the amount that the public is willing to pay is likely to widen. This may increase disparities for the patients who are able to pay and patients who are not able to pay. To mitigate this trend, the following should be considered. First, there is no consensus on the services that must be delivered by physicians, nor on the income level of physicians. Second, it is possible to set the income of primary care physicians so that it would be at the same or higher level as that of specialists. Thirdly, the listing of new pharmaceuticals in the government formulary could be made more transparent by evaluating patient outcomes with EQ-5D. Lastly, patients could be discouraged from visiting medical centers without referral by levying a copayment.

## Ethical issues

 Not applicable.

## Competing interests

 Author declares that he has no competing interests.
